# Endovascular management of unruptured intercostal artery aneurysms

**DOI:** 10.1186/s42155-018-0048-7

**Published:** 2019-01-04

**Authors:** Andrew Fenwick, Patrick Omotoso, Darren Ferguson

**Affiliations:** 1 Faculty of Medicine, Memorial University of Newfoundland, St. John’s, Newfoundland A1B 3V6 Canada; 2 Department of General Surgery, Dalhousie University, Saint John, New Brunswick Canada; 3 Department of Diagnostic Radiology, Dalhousie University, Saint John, New Brunswick Canada

**Keywords:** Intercostal artery, Aneurysm, Angiography, Endovascular procedure, Embolization

## Abstract

**Background:**

Intercostal artery aneurysms are rare vascular abnormalities that are typically diagnosed following rupture in patients with predisposing conditions. Our report is the first to document a patient with unruptured intercostal artery aneurysms in the absence of any associated disease.

**Case presentation:**

A 70-year-old male with prostatic adenocarcinoma was incidentally discovered to have multiple unruptured aneurysms of his intercostal arteries. Three of the aneurysms were embolized utilizing microcoils and glue. At six-month follow-up the patient remained asymptomatic.

**Conclusion:**

We demonstrate successful endovascular management of a unique case of multiple idiopathic unruptured intercostal artery aneurysms. Appropriate diagnosis and prompt treatment of these rare vascular lesions is essential in preventing the potentially catastrophic consequences of rupture.

## Background

Intercostal artery aneurysms are rare vascular abnormalities that are most often associated with aortic coarctation or neurofibromatosis type 1 (Tapping & Ettles, 2011; Dominguez et al., 2002). Aneurysm formation can also represent sequelae of vascular injury from systemic vasculitis, local infection, prior iatrogenic intervention, or previous trauma (Gonzalez et al., 2011; Bonne et al., 2015; Hernandez-Velasquez et al., 2016; Neuwirth & Singh, 2010). Patients with intercostal artery aneurysms are usually diagnosed following rupture, a potentially life-threatening complication (Arai et al., 2007; Kim et al., 2011). We describe a unique case of a patient who was incidentally found to have multiple unruptured intercostal artery aneurysms of indeterminate etiology. The clinical presentation, radiologic imaging, and endovascular management are discussed.

### Case presentation

**Fig. 1:**
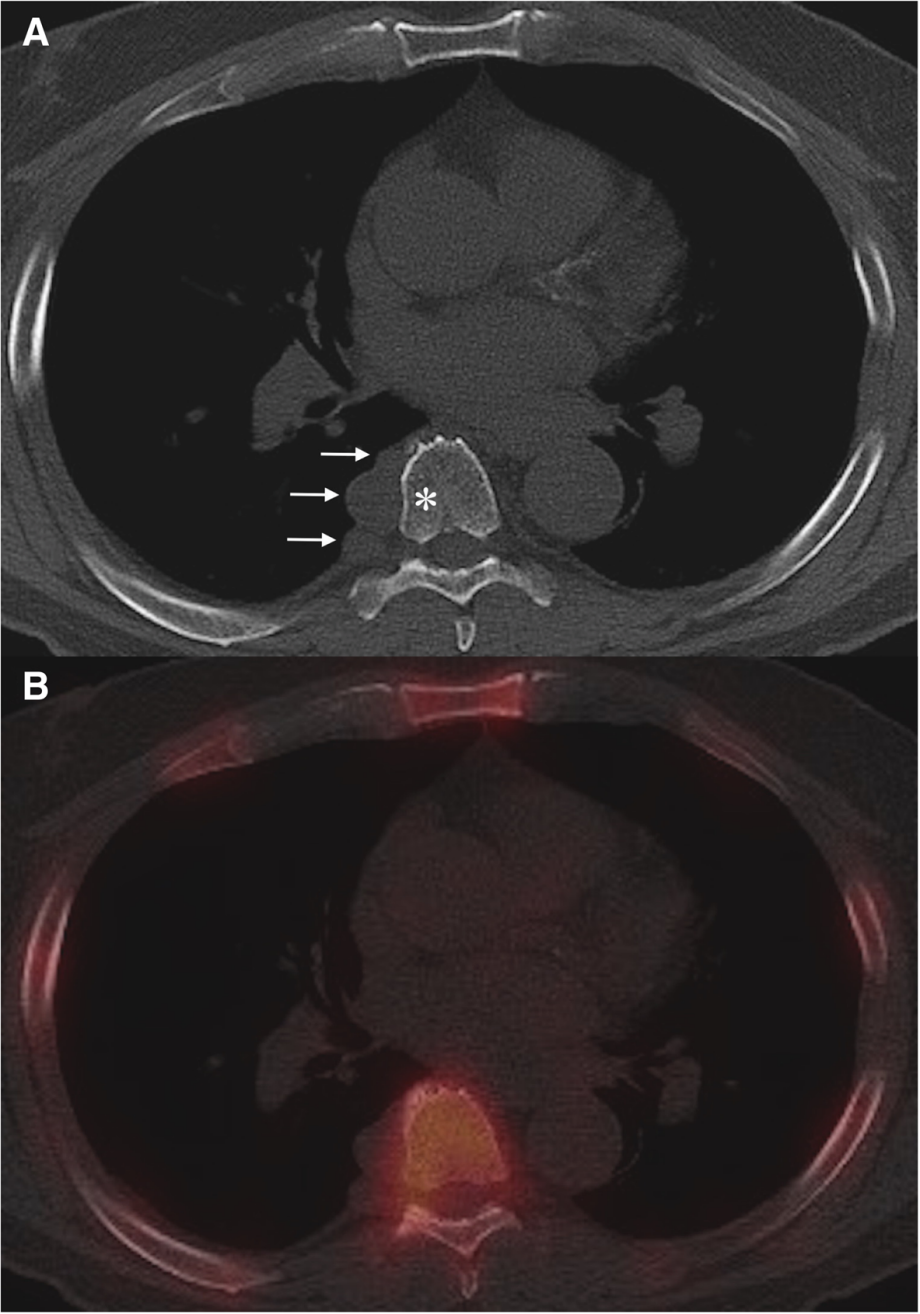
SPECT/CT 99mTc-MDP bone scan. **a** Axial CT image shows three extrapleural nodules (arrows) adjacent to the T7 vertebral body, which has a notched appearance (*). **b** Axial fused SPECT/CT image demonstrates no evidence of proximate bony metastatic disease

**Fig. 2:**
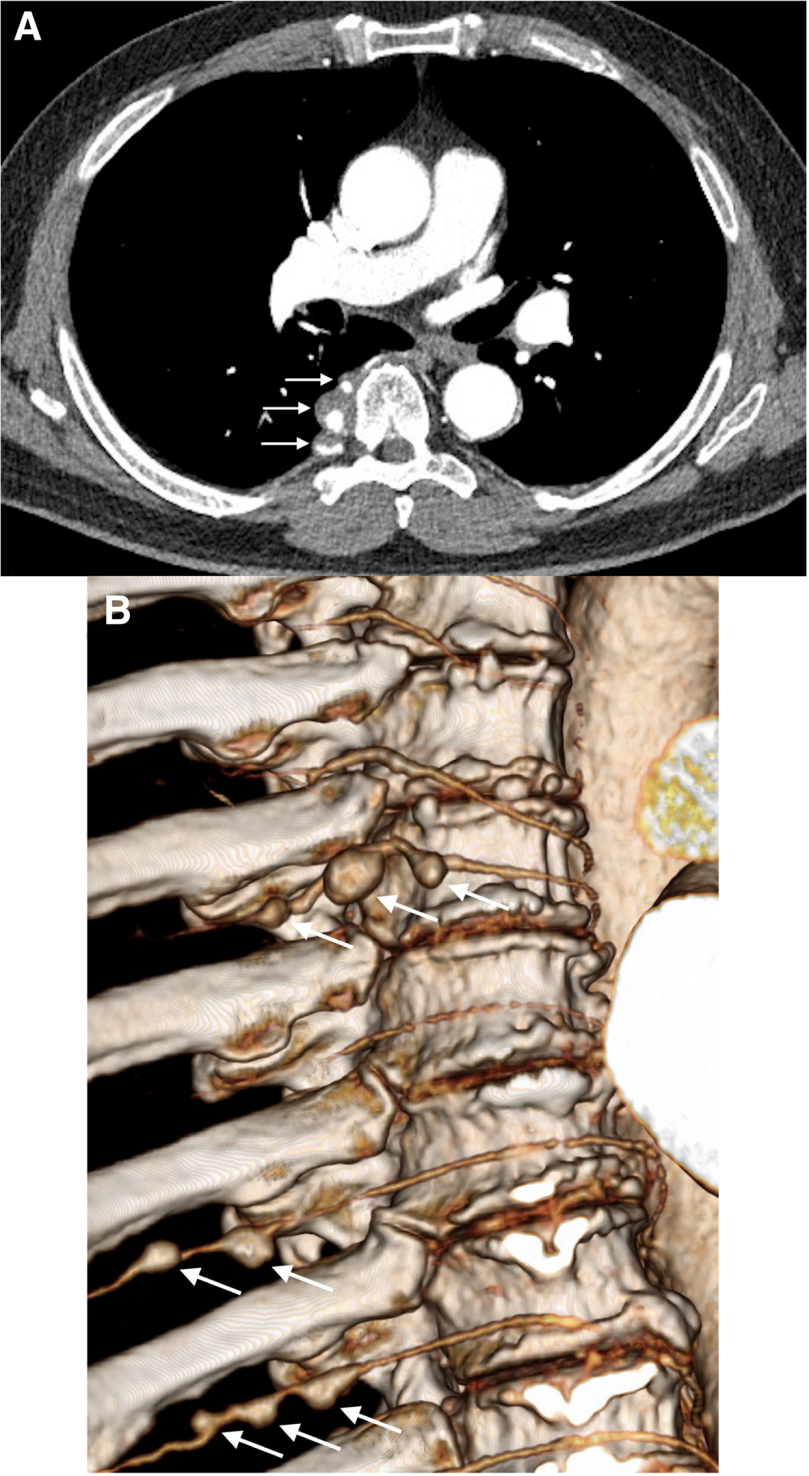
CT angiography. **a** Axial CT image shows three dense nodules and eccentric lower attenuation material (arrows) consistent with thrombus containing aneurysms of the proximal right 7th intercostal artery, the largest measuring 2.1 cm × 1.8 cm. **b** Three-dimensional reconstruction shows multifocal aneurysmal dilatations at multiple levels (arrows)

A 70-year-old male ex-smoker with hypertension, dyslipidemia, and newly diagnosed prostatic adenocarcinoma was undergoing staging prior to initiation of therapy. During this workup, a SPECT/CT scan noted vertebral body notching and multiple extrapleural nodules (Fig. 1). Further evaluation with CT angiography revealed multifocal saccular and fusiform aneurysms of the intercostal arteries (Fig. 2). No other aneurysms of the neck, chest, abdomen, or limbs were identified. It was decided to preventatively treat three large aneurysms of the right 7th intercostal artery with endovascular embolization.

**Fig. 3:**
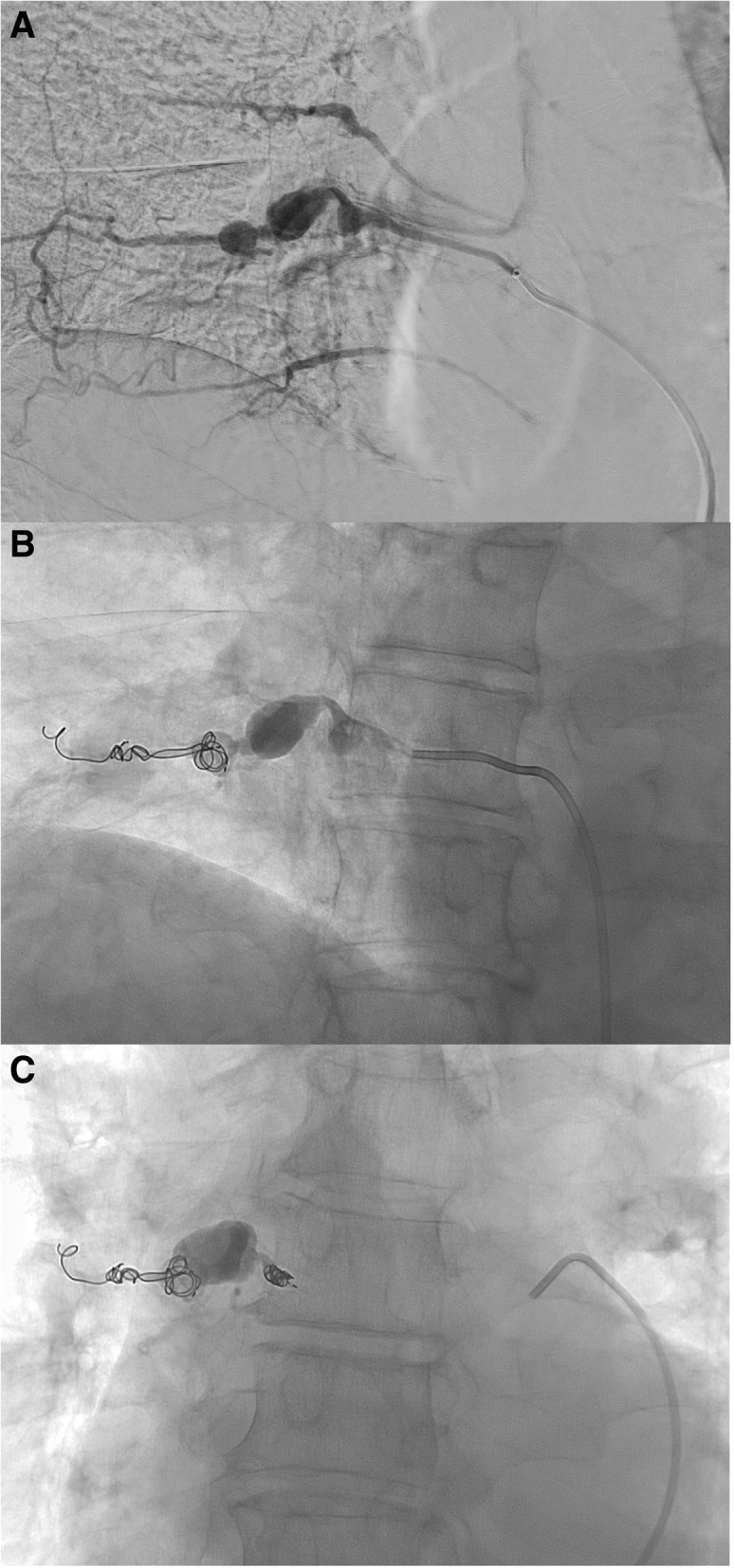
Selective intercostal angiography with digital subtraction. **a** Aneurysms of the right 7th intercostal artery prior to embolization, **b** after distal occlusion with microcoils, and **c** following complete exclusion of the aneurysm sacs with microcoils and glue

Following conscious sedation with Fentanyl and Midazolam and local anesthesia with 2% Lidocaine, the right common femoral artery was punctured utilizing a single-wall technique. A 6-Fr sheath was introduced and 5-Fr C2 Cobra catheter (Boston Scientific, Cork, Ireland) advanced selectively into the right 6th through 8th intercostal arteries. Angiography confirmed the target aneurysms of the 7th intercostal artery (Fig. 3a) and that no spinal artery originated from them. The 6th and 8th intercostal arteries did not provide significant collateral supply to the 7th intercostal artery. A Renegade microcatheter (Boston Scientific, Cork, Ireland) was inserted and Interlock microcoils (2 of 2 mm × 6 mm × 8 cm, Boston Scientific, Cork, Ireland) were deployed starting distally (Fig. 3b). To maximize the occlusive effect, the aneurysms were then embolized with a Glubran 2 (GEM, Viareggio, Italy)/Lipiodol (Guerbert, Roissy-en-France, France) mixture (1:1). Proximally, Interlock microcoils (2 of 2 mm × 4 mm × 4.1 cm, Boston Scientific, Cork, Ireland) were placed and complete cessation of flow was achieved (Fig. 3c). There were no intraoperative complications. The patient was discharged home the following day and made an uneventful recovery. At six-month follow-up, the patient remained asymptomatic and will be monitored with yearly CT angiograms.

## Discussion

The cause of intercostal artery aneurysm formation in this case remains unknown as no clinical or radiological evidence of any associated disease was identified. The presence of intercostal artery aneurysms in the absence of a predisposing condition is described in just two other case reports in the literature (Topel et al., 2004; Carr et al., 2013). As both of these patients presented with acute chest and back pain due to hemothorax, our report is the first to document idiopathic intercostal artery aneurysms prior to rupture. Thus, this is an important diagnosis to consider in patients with extrapleural nodules and associated rib or vertebral notching (Tapping & Ettles, 2011). A contrast-enhanced study may help to better characterize these lesions and assist with detection of additional aneurysms, which can involve multiple intercostal arteries (Carr et al., 2013).

There are no established criteria for the treatment of intercostal artery aneurysms. In this case we felt that intervention was justified to reduce the likelihood of aneurysm rupture, thoracic hemorrhage, and sudden death (Dominguez et al., 2002; Hernandez-Velasquez et al., 2016; Arai et al., 2007; Kim et al., 2011; Topel et al., 2004; Carr et al., 2013; Aizawa et al., 2010). Endovascular management of unruptured intercostal artery aneurysms has shown success in a small number of patients to date (Tapping & Ettles, 2011; Bonne et al., 2015; Neuwirth & Singh, 2010; Uzuka et al., 2012). Similar to the technique described by Bonne et al. (2015), we decided on a minimally invasive approach with selective embolization combining microcoils and glue rather than a thoracotomy with ligation, clipping, or excision. Aneurysms are fragile and minimizing the potential for complications such as paraplegia from post-operative hematoma compression is crucial (Aizawa et al., 2010), especially when the aneurysms are in close proximity to the spinal cord as they were in our patient. Since the Adamkiewicz artery did not arise from the target intercostal artery, embolization of the largest aneurysm and two adjacent aneurysms was safely performed. We elected not to treat the other unruptured aneurysms given their sub-centimeter size and location in the lower intercostal arteries where the Adamkiewicz artery most commonly originates. Close follow-up with annual CT angiograms will be used to ensure durable occlusion and to assess for disease progression. Any aneurysms increasing to ≥1 cm in size will be discussed at vascular rounds to weigh the potential benefits of embolization versus the possible risks of complications.

## Conclusion

This report demonstrates a unique case of a patient with multiple idiopathic unruptured intercostal artery aneurysms that were successfully managed with endovascular embolization. Appropriate diagnosis and prompt treatment of these rare vascular lesions is essential in preventing the potentially catastrophic consequences of rupture.
