# Correction to: Endovascular management of unruptured intercostal artery aneurysms

**DOI:** 10.1186/s42155-020-0101-1

**Published:** 2020-03-16

**Authors:** Andrew K. C. Fenwick, Patrick Omotoso, Darren Ferguson

**Affiliations:** 1 Faculty of Medicine, Memorial University of Newfoundland, St. John’s, Newfoundland A1B 3V6 Canada; 2 Department of General Surgery, Dalhousie University, Saint John, New Brunswick Canada; 3 Department of Diagnostic Radiology, Dalhousie University, Saint John, New Brunswick Canada

**Correction to: CVIR Endovasc**

**https://doi.org/10.1186/s42155-018-0048-7**

‘In the published article (Fenwick et al., 2019) the statement under the subheading ‘Consent for publication’ is incorrect.

“Waived by Institutional Review Board for case reports.”

should read:

“Informed consent for publication of this case report and its accompanying images has been obtained from the patient”.
